# Melanocytic lesions of the central nervous system: a case series

**DOI:** 10.1590/0004-282X-ANP-2021-0082

**Published:** 2022-03-15

**Authors:** Jorge VARELA-POBLETE, Aaron VIDAL-TELLEZ, Juan Pablo CRUZ-QUIROGA, Francisca MONTOYA-SALVADORES, Jaime MEDINA-ESCOBAR

**Affiliations:** 1 Universidad de Valparaíso, Instituto de Neurocirugía Dr. Asenjo, Departmento de Neurorradiología Diagnóstica, Santiago, Chile. Universidad de Valparaíso Instituto de Neurocirugía Dr. Asenjo Departmento de Neurorradiología Diagnóstica Santiago Chile; 2 Universidad de Chile, Hospital del Salvador, Departamento de Neurología, Santiago, Chile. Universidad de Chile Hospital del Salvador Departamento de Neurología Santiago Chile

**Keywords:** Central Nervous System Diseases, Melanosis, Neurocutaneous Syndromes, Magnetic Resonance Imaging, Neoplasm Metastasis, Enfermedades del Sistema Nervioso Central, Melanosis, Síndromes Neurocutáneos, Imagen por Resonancia Magnética, Metástasis de la Neoplasia

## Abstract

**Background:**

Melanocytic lesions of the central nervous system (CNS) are an infrequent, broad and diverse group of entities, both benign and malignant, found in all age groups, with imaging findings ranging from well-circumscribed focal lesions to diffuse leptomeningeal involvement. On MRI, they are usually distinguished by a high signal on T1WI sequences, given the paramagnetic effect of melanin, thus making it difficult to differentiate among them.

**Objective:**

To describe the imaging and epidemiological characteristics of a retrospective series of CNS melanocytic lesions.

**Methods:**

MR images of 23 patients with CNS melanocytic lesions diagnosed between January 2012 and June 2018 were analyzed.

**Results:**

Most patients were female (14/23; 61%), with a median age of 47 years (range: 3 weeks to 72 years). The primary melanocytic lesions accounted for 8/19 cases (42.1%), which included neurocutaneous melanosis, meningeal melanocytomas and primary malignant melanomas. Secondary melanocytic lesions (metastatic) accounted for 10/19 cases (52.6%). There was one case of a tumor with secondary melanization, from a melanocytic neuroectodermal tumor of infancy. There were also four cases of primary ocular melanomas. The most frequent findings were the cerebral location, high T1WI signal and marked contrast-enhancement.

**Conclusions:**

The present review describes the wide variety of melanocytic lesions that could affect the CNS, emphasizing the MRI characteristics. Knowledge of the imaging, clinical and epidemiological characteristics of CNS melanocytic lesions is essential for their correct interpretation, given the significant overlap between lesion features and the variable prognosis.

## RESUMEN

**Antecedentes:**

Las lesiones melanocíticas del sistema nervioso central (SNC) corresponden a un grupo infrecuente, amplio y diverso de entidades, tanto benignas como malignas, encontradas en todos los grupos etarios, con hallazgos imagenológicos que van desde lesiones focales bien circunscritas hasta un compromiso leptomeníngeo difuso. A la RM se distinguen por la alta señal en la secuencia T1WI, dado el efecto paramagnético de la melanina, haciendo difícil la diferenciación entre ellas.

**Objetivo:**

Describir las características epidemiológicas y de de una serie retrospectiva de lesiones melanocíticas del SNC.

**Métodos:**

Revisión de imágenes de RM de 23 pacientes con lesiones melanocíticas del SNC diagnosticadas entre enero de 2012 y junio de 2018.

**Resultados:**

La mayoría de los pacientes fueron mujeres (14/23; 61%), con edades comprendidas entre las 3 semanas de vida hasta los 72 años. Las lesiones melanocíticas primarias representaron 8/19 (42,1%), incluyendo: melanosis neurocutáneas, melanocitomas meníngeos y melanomas malignos primarios. Las lesiones melanocíticas secundarias (metastásicas) representaron 10/19 casos (52,6%). Hubo un caso de tumor con melanización secundaria (tumor neuroectodermico melanocítico de la infancia). Se incluyeron cuatro casos de melanomas oculares primarios. Los hallazgos más frecuentes fueron la localización cerebral, el aumento de señal T1 y el acentuado realce con el gadolinio.

**Conclusiones:**

Se describe la amplia variedad de lesiones melanocíticas encontradas en el SNC, enfatizando sus características a la RM. El conocimiento de sus características imagenológicas, clínicas y epidemiológicas es fundamental para su correcta interpretación, dado la notable superposición entre las presentaciones de las lesiones y lo variable de sus pronósticos.

## INTRODUCTION

Melanocytic lesions of the central nervous system (CNS) are an infrequent and varied group of entities that range from benign to malignant lesions and from focal to diffuse leptomeningeal involvement. Their imaging appearance will depend largely on their intrinsic melanin content. They are usually classified into primary, secondary (metastases) and tumors with secondary melanization (schwannomas, medulloblastomas, some glial tumors and melanocytic neuroectodermal tumors of infancy)^1^^,^^2^^,^^3^.

Primary melanocytic lesions are very rare, with an estimated incidence of 0.9 per 10 million people/year^2^. The latest CNS tumor classification from the World Health Organization (WHO), of 2016, includes them in the chapter “Tumors of the Non-Meningothelial Mesenchyme” and groups them into four entities: i) meningeal melanocytosis; ii) meningeal melanomatosis (often occurring in the context of dermatological syndromes, called neurocutaneous melanosis); iii) meningeal melanocytoma; and iv) meningeal melanoma^3^. However, the most frequent CNS melanocytic lesions are secondary or metastatic. Metastatic melanoma is the third most frequent source of brain metastases after lung and breast cancer^1^^,^^2^.

Previous studies are scarce and generally limited to reports and case series, thus making diagnostic and management standardization difficult for these patients^4^.

The purpose of our study was to review the epidemiological characteristics and MRI features of CNS melanocytic lesions, through a retrospective study on a case series.

## METHODS

We conducted a descriptive retrospective study on patients with melanocytic lesions of the CNS who were identified through searching the imaging databases at our institution covering the period from January 2012 to June 2018. As this was a retrospective study, ethics committee approval and informed consent were waived.

The search was carried out among MRI reports, and those in which it was concluded that some type of CNS melanocytic lesion was present were selected. The search terms used were: “melanocytosis”, “melanocytoma, “melanoma”, “melanomatosis”, “melanocytic”, “melanocytic lesions”, “melanosis” and “melanization”. Subsequently, clinical and pathological data were collected when available.

Although primary ocular melanomas are not considered in the usual classifications of CNS melanocytic lesions, they were included here, given their relative high frequency in adults, with prognostic importance and distinctive imaging characteristics^5^.

The MRI studies that were searched here had been performed in 3T equipment (Magnetom Skyra, Siemens, Medical Systems, Erlanger, Germany) and in 1.5T equipment (Intera, Philips Medical Systems, Best, Netherlands). All MRI protocols included the following sequences: conventional T1/T2-weighted imaging (T1/T2WI); susceptibility-weighted (T2*/SWI); diffusion-weighted (DWI/ADC maps) and contrast-enhanced T1 (T1WI-Gd). The information on all available MRI studies was collected from the radiological reports and biopsies were interpreted by certified neuropathologists (C.T.G. and S.C.B).

The features studied were the location and imaging aspects of the lesions. The imaging appearance was analyzed in the T1WI, T2WI, T1WI-Gd and DWI/ADC sequences. Analyses on gradient (GRE) or magnetic susceptibility (SWI) sequences were excluded, given the different magnets used and the difficulty in differentiating imaging between melanin and methemoglobin, given their similar paramagnetic effects^1^^,^^6^.

Hyperintense lesions in T1WI were considered to be present when they presented higher signal intensity than normal brain cortex in some component of the lesion. Similarly, in T2WI sequences, the lesions were considered to be hyperintense, isointense or hypointense, according to their predominant signal, in relation to the signal intensity of the cortex. Regarding T1WI-Gd sequences, contrast enhancement was characterized as present (“+”) or absent (“-”) and according to its distribution, as focal, meningeal or mixed. Similarly, restricted diffusion was considered to be present when the ratio of ADC values was lower than that of the pontine white matter, in the solid component of the lesion.

## RESULTS

A total of 32 patients were found in our report database. Nine patients were excluded because no preoperative MRI was available. Thus, 23 patients were included for the analysis: 14 females and 9 males, with a median age of 47 years (range: 3 weeks to 72 years). There were 18 adults and 5 pediatric patients. In 19 patients, the tumors were described as a CNS melanocytic lesion and in four as a primary ocular melanoma. The compartmental location of the lesions is described in Table 1.

**Table 1.** Compartmental location of central nervous system melanocytic lesions.

CNS location	Compartment
Brain (17/19; 89.4%)	Supratentorial only (8/19; 42.1%) Parenchymal-meningeal (7/19; 36.8%) Intraventricular (1/19; 5.2%) Infratentorial only (4/19; 21%)
Spinal (2/19; 10.5%) (one vertebral body T9; one intramedullary T2)	Both supra+infratentorial (5/19; 26.3%)
Ocular (4)	

CNS: central nervous system.

Primary melanocytic lesions accounted for 8/19 cases (42.1%): 4 pediatric and 4 adult cases, with a median age of 43 years (range: 3 weeks to 67 years). Secondary melanocytic lesions accounted for 10/19 cases (52.6%), with a median age of 51 years (range: 34-72 years). The only case of a tumor with secondary melanization had an age at diagnosis of 2 months. The primary ocular melanomas comprised 4 cases, with ages ranging from 41 to 64 years, and a median of 42 years. The specific diagnoses, availability of histological confirmation, ages and imaging characteristics of the primary and secondary melanocytic lesions and primary ocular melanomas are described in Tables 2, 3 and 4, respectively.

**Table 2.** Distribution of primary melanocytic lesions and their imaging characteristics.

No.	Age	Sex	Diagnosis	Biopsy	Location	T1 signal	T2 signal	T1Gd	Restricted diffusion
1	3 weeks	F	Neurocutaneous melanosis	(-)	Supra-and infratentorial (meningeal+parenchymatous)	Hyper	Hypo	+;mixed*	(-)
2	2 years	M	Neurocutaneous melanosis	(-)	Supra-and infratentorial (meningeal)	Hyper	Iso	+; Meningeal	(-)
3	3 months	M	Neurocutaneous melanosis	(-)	Supra-and infratentorial (meningeal)	Hyper	Iso	+; Meningeal	(-)
4	5 months	M	Neurocutaneous melanosis	(-)	Supra-and infratentorial (meningeal+parenchymatous)	Hyper	Hypo	+; mixed	(-)
5	67 years	M	Melanocytoma	(-)	Spinal (dorsal cord)	Hyper	Hyper	+; focal	(-)
6	43 years	M	Melanocytoma	(-)	Infratentorial	Hyper	Hypo	+; focal	(-)
7	46 years	F	Primary malignant melanoma	(+)	Intraventricular	Hyper	Hypo	+; focal	(+)
8	41 years	M	Primary malignant melanoma (amelanocytic)	(+)	Infratentorial	Hypo	Iso	+; focal	(-)

M: masculine; F: feminine; (-): absent; (+): present; Hyper: hyperintensity; Hypo: hypointensity; Iso: isointensity; *mixed: both meningeal and focal enhancement.

**Table 3.** Distribution of secondary melanocytic lesions and their imaging characteristics.

No.	Age(*)	Sex	Diagnosis	Biopsy	Location	No. of lesions	T1 signal	T2 signal	T1Gd	Restricted diffusion
1	48	F	MTT melanoma-amelanocytic	(+)	Supratentorial	2	Hypo	Hypo/iso	+; focal	(-)
2	72	M	MTT melanoma	(+)	Supratentorial	2	Hyper	Hypo	+; focal	(-)
3	50	F	MTT melanoma	(+)	Supra-and infratentorial	6	Hyper	Hypo	+; focal	(-)
4	47	F	MTT melanoma	(+)	Supratentorial	2	Hyper	Iso	+; focal	(+)
5	43	M	MTT melanoma	(+)	Supratentorial	1	Hyper	Hypo	+; focal	(-)
6	52	F	MTT melanoma	(+)	Infratentorial	1	Hyper	Hypo	+; focal	(-)
7	57	F	MTT melanoma	(-)	Supratentorial	1	Hyper	Hypo	+; focal	(-)
8	67	F	MTT melanoma	(-)	Supratentorial	1	Hyper	Hypo	+; focal	(-)
9	56	F	MTT melanoma	(-)	Infratentorial	2	Hyper	Hypo	+; focal	(-)
10	34	F	MTT melanoma	(+)	Supratentorial-spinal	2	Hyper	Hypo	+; focal	(-)

M: masculine; F: feminine; (-):absent; (+): present; Hyper: hyperintensity; Hypo: hypointensity; Iso: isointensity; (*): in years; MTT: metastatic.

**Table 4.** Distribution of primary ocular melanomas and their imaging characteristics.

No.	Age (*)	Sex	Diagnosis	Biopsy	Location	T1 signal	T2 signal	T1Gd	Restricted diffusion
1	64	F	Ocular melanoma	(-)	Left orbit -posterosuperior wall	Hyper	Hypo	+; focal	(+)
2	41	M	Ocular melanoma	(-)	Left orbit -medial wall	Hyper	Hypo /iso	(-)	(-)
3	41	F	Amelanocytic ocular melanoma	(-)	Left orbit -posterosuperior wall	Iso	Hypo /iso	+; focal	(+)
4	43	F	Ocular melanoma	(-)	Left orbit -posterosuperior wall	Hyper	Hyper	+; focal	(-)

M: masculine; F: feminine; (-): absent; (+): present; Hyper: hyperintensity; Hypo: hypointensity; Iso: isointensity; (*): in years.

**Figure 1.**
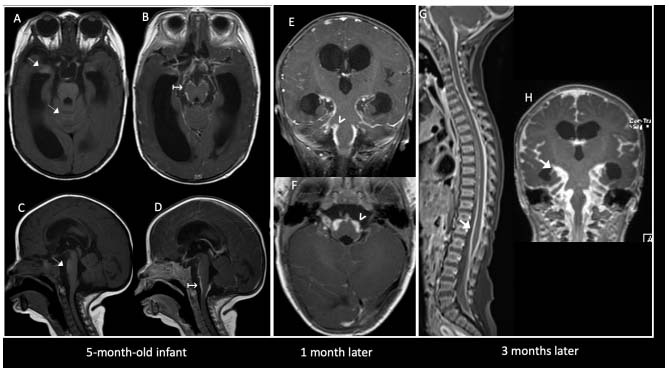
Neurocutaneous melanosis. A 5-month-old infant, without pigmented skin lesions, who was referred due to acute hydrocephalus. Axial (A, B) and sagittal (C, D) pre and post-contrast T1WI demonstrated subtle foci of spontaneous hypersignal in the temporal uncus, brainstem and cerebellar vermis (thin white arrows), with extensive leptomeningeal enhancement (↦). In a control one month later, progression of leptomeningeal enhancement was observed, which was predominantly infratentorial (arrowheads in E, F). A control three months later (G, H) showed aggressive spinal and encephalic progression of leptomeningeal dissemination (thick white arrows).

Neurocutaneous melanosis accounted for 50% (4/8) of all the primary melanocytic lesions (Figure 1), and most of them occurred in infants younger than 6 months old. Lesions were located both supra and infratentorially, predominantly in the anterior temporal lobes and brainstem. Three of the four cases had concomitant skin nevi. Histological confirmation was not available in this group. The only case without skin lesions evolved with aggressive clinical and imaging behavior (thick and irregular leptomeningeal enhancement and signs of perivascular invasion)^1^^,^^2^. All of these cases were hyperintense on T1WI and showed diffuse leptomeningeal nodular enhancement. There was no restricted diffusion.

**Figure 2.**
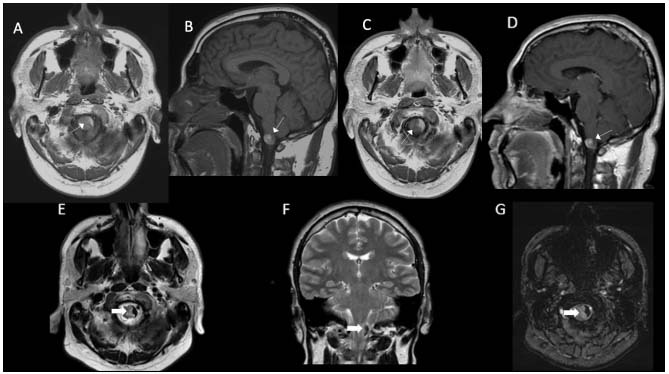
Melanocytoma. A 43-year-old man was referred for assessment of a cervicomedullary lesion. Axial and sagittal T1WI pre-contrast (A, B) and post-contrast (C, D) showed a spontaneously hyperintense lesion in the left lateral aspect of the cervicomedullary junction, abutting the pial surface (thin white arrows), with subtle heterogeneous enhancement (↦). T2WI axial and sagittal (E, F) showed marked hypointensity with a slight susceptibility artifact in the SWI sequence (G) (thick white arrows).

There were two cases of primary meningeal melanocytomas (Figure 2), both males, with lesions in the dorsal cord and at the cervicomedullary junction. Both were hyperintense on T1WI. In T2WI they presented heterogeneous signals, with hyperintense predominance in the former and hypointense in the latter. Histological confirmation was not carried out in either of these cases.

**Figure 3.**
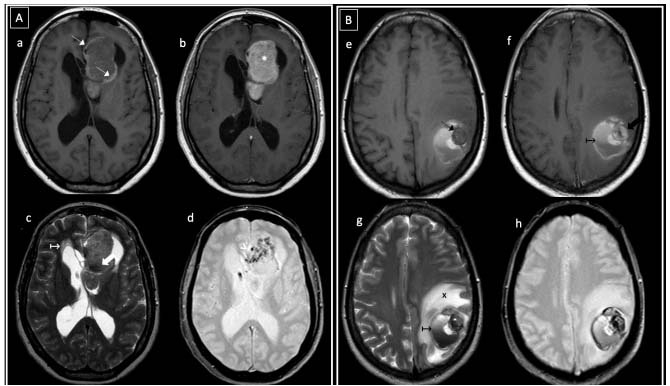
Intraventricular primary melanoma (A) and amelanocytic metastatic melanoma (B). In (A), a 46-year-old woman presented with headache. Axial T1WI pre and post-Gd (a, b) showed a heterogeneous left frontal intraventricular mass, with peripheral high signal areas (thin white arrows) and marked contrast enhancement (* in b). T2WI and GRE (c, d) showed hypointense foci (thick white arrows) and intratumoral magnetic susceptibility artifacts (white arrowhead). No restricted diffusion was observed (not shown). Hydrocephalus was present (white ↦ in c). In (B), a 48-year-old woman with a history of cutaneous melanoma presented with seizures. Axial MRI images (e-h) showed a hypointense-T1WI left parietal intra-axial peripheral nodule (thin black arrow in e) with heterogeneous contrast enhancement (thick black arrow in f). The lesion was surrounded by hemorrhagic products and hematocrit level (black ↦). Extensive vasogenic edema was observed (black x in g).

There were two cases of primary malignant melanomas (Figure 3A): one intraventricular and the other infratentorial (cervicomedullary junction), aged 46 and 41 years, respectively. In both cases, there was histological confirmation and exclusion of primary melanoma outside the CNS.

The secondary melanocytic lesion group (metastatic melanomas) showed several lesions in most of the cases, ranging from two to six lesions. Brain location predominated (9/10; 90%), while there was a single case with spinal (vertebral) involvement. In 60% there was an exclusive supratentorial location and in 20% an exclusive infratentorial location. One case was both supra and infratentorial and one was both supratentorial and vertebral. Most of the cases presented the classic melanocytic pattern of high T1WI signal intensity, while in a single one case a supratentorial amelanocytic lesion was observed (Figure 3B). Focal contrast enhancement was observed in all cases and restricted diffusion was demonstrated in only one case.

**Figure 4.**
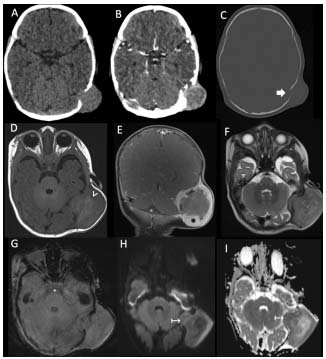
Melanocytic neuroectodermal tumor of infancy, in a two-month-old infant who presented with a mass over the left mastoid fontanelle. Axial pre and post contrast CT scans (A-C) showed an apparently extracranial mass at the level of the left mastoid fontanelle, with peripheral enhancement (thin white arrow in B) and osteolytic and remodeling bone changes (thick white arrow). MRI images (D-I) demonstrated a predominantly extracranial heterogeneous mass with intra and extracranial components, hyperintense areas on T1WI (white arrowhead in D) and avid peripheral contrast enhancement (* in coronal image in E). No magnetic susceptibility artifacts were observed on SWI (G). In DWI and ADC (H and I), some deep areas of restricted diffusion were presented (↦).

In the group of “tumors with secondary melanization”, a single case of a two-month-old male infant with a mass over the left mastoid fontanelle was found. A biopsy on this mass showed it to be a melanocytic neuroectodermal tumor (Figure 4).

**Figure 5.**
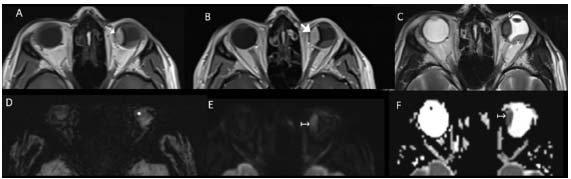
Left choroidal ocular melanoma, in a 41-year-old male patient with a history of progressive blurred vision in the left eye. Axial MRI images (A-F) showed a left intraocular fusiform lesion in the choroidal retinal nasal wall, with spontaneously hyperintense signal on T1WI (thin white arrow in A), with no apparent contrast enhancement (thick white arrow in B). There was low signal intensity on T2WI (white arrowhead in C), a susceptibility artifact on SWI sequence (* in D) and restricted diffusion (↦ in E and F). The melanoma was also associated with retinal detachment (x in C).

Primary ocular melanomas (Figure 5) were located in the left ocular globe, and mostly in the posterosuperior wall. There was retinal detachment in all cases. The imaging findings were mainly high T1WI signal intensity and focal contrast enhancement, with a single case of amelanocytic melanoma. In two cases restricted diffusion was observed.

## DISCUSSION

Melanocytes are the cells that produce and store melanin. They originate from the neural crest and, between the 8^th^ and 10^th^ weeks of embryonic development, they migrate to the skin, mucosa, uveal ocular layer, inner ear and CNS. In the latter, they are located in the leptomeninges, where they are preferentially distributed in the convexity of the skull base, ventral brainstem and cervical cord^2^^,^^7^.

Melanin has a paramagnetic effect, which induces shortening of the T1 and T2 relaxation times on MRI. This gives rise to its characteristic high signal intensity in T1WI, with corresponding hypointensity in T2WI^2^. It is important to highlight that lesions with a melanin content greater than 10% will present the classic “melanocytic” pattern of T1WI hyperintensity/T2WI hypointensity signal on MRI. Lesions that appear “amelanocytic” have in fact been shown to contain some degree of melanin, in histopathological evaluations^1^.

In our retrospective series, a total 23 cases were identified, with predominance of the female sex, in contrast to the slight male predominance previously reported^1^^,^^5^^,^^7^. There was a wide age range at presentation (3 weeks to 72 years), with some overlap between the different diagnoses, similar to what had previously been reported^6^^,^^8^.

The location of the melanocytic lesions was consistent with what had been reported^1^^,^^2^, with clear predominance of the brain over the spine. Most of these lesions were supratentorial (parenchymal-meningeal and intraventricular), followed by a combination of both supra and infratentorial and lastly, infratentorial involvement alone. We decided to include the intraocular melanomas, given their incidence, prognosis and distinctive imaging features, which allow them to be differentiated from other entities.

Regarding the imaging characteristics, the classical melanocytic pattern of T1WI hyperintensity/T2WI hypointensity signal predominated. In three cases (metastatic melanoma, ocular melanoma and primary malignant melanoma), an “amelanocytic” pattern was observed, with ­intermediate/­low T1WI signals. Almost all the cases presented contrast enhancement, either focal, leptomeningeal or mixed, with the exception of one case of ocular melanoma with a highly melanocytic pattern. These characteristics were the most constant imaging markers described^1^^,^^2^.

Primary malignant melanomas of the CNS are very infrequent, with estimated annual incidences of 0.7 per 10 million inhabitants^9^. They occur in adults, with a mean age at presentation of 50 years, and are located in the basal leptomeninges, around the brainstem and upper cervical cord. Their imaging appearance will depend on the melanin and methemoglobin content from prior bleeding episodes^1^^,^^2^^,^^7^^,^^9^^,^^10^. Their diagnosis is based on the absence of any other melanoma, both outside the CNS and in other CNS sites^1^. The two cases in our series consisted of a cervicomedullary junction “amelanocytic” melanoma and an intraventricular “melanocytic” melanoma. The latter has been described as an atypical site and would be explained by arrested migration of melanocytic cells, through which they are deposited within the pia mater. From their location within the pia mater, melanocytes may be incorporated into the choroid plexus^1^.

Meningeal melanocytomas, previously called melanocytic meningiomas, are very rare benign lesions, with an estimated annual incidence of one case per million inhabitants, with isolated reports of malignant transformation. They also occur predominantly in the fifth decade of life and they tend to be located in the posterior fossa, Meckel’s cavum and cervicothoracic spinal canal^1^^,^^11^. They are well-defined solitary lesions, with close leptomeningeal contact, variable hyperintense T1WI/hypointense T2WI signal and avid homogeneous enhancement^12^^,^^13^^,^^14^. Our two cases had peripheral cervicomedullary junction and dorsal medullary locations, concordant with what had previously been reported.

Neurocutaneous melanosis is a rare type of non-inherited phacomatosis, characterized by congenital cutaneous nevi and leptomeningeal melanocytic proliferation. Around 100 cases have been reported in the literature^1^^,^^15^. They were believed to be the result of congenital dysplasia of the melanocyte precursor cells in the neuroectoderm, which would cause abnormal proliferation of melanocytes in the skin and leptomeninges^15^^,^^16^. There is an association with the Dandy-Walker malformation in 8 to 10% of the cases^1^ and these usually debut with hydrocephalus, related to obstruction of CSF ​​flow or impaired reabsorption secondary to meningeal involvement. Neurological manifestations derive from intracranial hypertension and generally occur before the age of two years^15^. The imaging study of choice is MRI, in which two patterns can be observed: one of diffuse enhancement of thickened leptomeninges and a second of meningeal disease with characteristic high signal intensity on T1WI, most evident in the anterior temporal lobes (as shown in Figure 1A) and amygdala region, followed by the cerebellum, protuberance, thalamus and frontobasal parenchyma^1^^,^^15^. Malignant transformation is reported in approximately 40-60% of cases, and is indicated by progressive growth, surrounding vasogenic edema or mass effect, or development of central necrosis^1^^,^^2^^,^^15^^,^^17^. In our series, we found four cases, all under two years of age and all with diffuse supra and infratentorial leptomeningeal involvement and hydrocephalus. Two of them evolved aggressively, with greater extent and severity of lesions.

Secondary melanocytic lesions (metastatic melanoma) were the most frequently found diagnosis, similar to what had previously been reported^2^. The CNS has been described as a frequent location, and is the third after lung and breast neoplasms^1^. An even higher incidence has been described in cases in which the primary site of malignant melanoma manifests between the ages of 50-59 years^18^. These lesions present a wide variety of appearances and locations. They usually debut as several lesions and the brain is the most common location followed by the cerebellum^1^. They typically appear at the peripheral gray matter-white matter junction, with miliary and subependymal distribution patterns and some rare cases have been reported in the choroid plexus and pituitary gland^19^. Imaging appearances are quite variable, ranging from the classic “melanocytic” pattern of T1WI ­hyperintensity/­T2WI hypointensity signal, up to the “amelanocytic” pattern. Marked contrast enhancement and peripheral edema are characteristic. Variable degrees of melanin and methemoglobin are presented, and the latter is derived from the tendency to bleed. However, there is no consensus on whether the image appearance depends on one of these compounds or on both^1^^,^^19^. The majority of our cases presented several lesions (between two and six), with an exclusive brain location predominance, “melanocytic” pattern and marked contrast enhancement.

Regarding the third group of CNS melanocytic lesions, i.e. tumors with secondary melanization, we presented a single case of an infant with a melanocytic neuroectodermal tumor of infancy. These are very rare lesions that affect newborns and children under one year of age, with a certain male predominance^1^^,^^8^^,^^20^. These lesions are of unknown etiology and histogenesis, and a probable origin in neural crest cells has been proposed^1^^,^^8^. Although some authors have included them within “neoplasms that undergo melanization”^1^, other authors have included them within primary melanocytic lesions^2^. They have rapid growth, with malignant transformation rates reported between 6.5 and 14.3%^8^. They are described as well-defined round or lobulated contrast-enhancing tumors, with areas of T1WI hyperintensity/T2WI hypointensity signal, mostly located in the maxillary bone (60% of cases) and skull (10.8%), especially in the region of the anterior fontanel, dura and brain^1^^,^^20^. Our only case was a two-month-old male patient, who had a palpable mass in the left parieto-occipital region, at the level of the mastoid fontanelle, with features similar to those reported in the literature.

Lastly, primary ocular melanoma is the most frequent malignant intraocular tumor in adults, with an incidence of approximately six cases per million/year^5^. It typically originates from the uveal layer of the eye (ciliary body, iris and, especially, the choroid), although a few articles have reported a conjunctival source^5^^,^^21^. It is the second location of primary melanoma after cutaneous melanoma and although the vast majority are primary, ocular melanoma can also have metastatic origin from a distant skin lesion^22^. Its incidence increases with age, with a peak in the 7^th^ to 8^th^ decade of life. It may or may not be symptomatic (visual defects and photopsia). On MRI, it presents lentiform or mushroom-shaped morphology, with a broad base of choroidal implantation and protrusion to the vitreous chamber, usually associated with retinal detachment^22^. It typically presents with the classic “melanocytic” pattern of T1WI hyperintensity/T2WI hypointensity signal, although an amelanocytic pattern may exist in up to 25% of the cases. It usually presents marked solid contrast enhancement and restricted diffusion, and the latter differentiates it from retinal detachment due to other causes^22^. The imaging features of our four cases were concordant with what had been reported in the literature, i.e. choroidal location, posterosuperior wall predominance and all associated retinal detachment. Three cases presented diffuse contrast enhancement with gadolinium, two cases restricted diffusion and one case an “amelanocytic” pattern.

In conclusion, CNS melanocytic lesions are rare and can be found in all age groups. The imaging characteristics are miscellaneous, depending on each specific diagnosis, its location and the amount of melanin contained. Nevertheless, there are some MRI features that can suggest this kind of lesions, like the high signal intensity in T1WI, low signal intensity in T2WI, restricted diffusion and either nodular or leptomeningeal contrast enhancement. In many cases, the diagnostic approach will be challenging, given the low incidence and frequent overlap of patterns. When faced with a suspicious lesion, it is essential to know the wide differentials like those described here, especially the feared metastatic melanoma, given its greater frequency and poor prognosis.
